# Supplementary material for: Improved genetic algorithm based on greedy and simulated annealing ideas for vascular robot ordering strategy
Source: PLoS One. 2025 Feb 20;20(2):e0306990. doi: 10.1371/journal.pone.0306990 (PMC11841910; doi:10.1371/journal.pone.0306990)
Supplement: S1 File — (DOCX) [file pone.0306990.s002.docx]

Highlights :

- Comprehensive Resource Allocation Model: We have developed a robust resource allocation model that optimizes the procurement of both robotic vessels and operators, considering the dynamic nature of healthcare environments.
- Incorporating Adaptive Learning: Our model accounts for the adaptive learning process required for operators, as well as the maintenance and disposal of robotic components.
- Hybrid Genetic Algorithm: We introduce a hybrid genetic algorithm that incorporates simulated annealing and greedy approaches to efficiently solve the optimization problem.
- Time Series Forecasting: We use an ARIMA time series model to predict the demand for vascular robots, enhancing the adaptability of our procurement strategy.
